# New York Pathological Society

**Published:** 1859-11

**Authors:** E. Lee Jones


### ART. II.—*New York Pathological Society.*

Regular Meeting, Sept. 14, 1859. John C. Dalton, Jr., M.D., President.

(Reported for the Journal by E. Lee Jones, M. D., Secretary.)

### CONGENITAL HERNIA OF THE FUNIS.

Dr. Briddon presented a specimen, with the following history.

On the 9th of July, 1859, I saw the male child of Hugh and Mary O'Connor, in company with Drs. Pulling, Aigner, and William Corson. The patient was three days old, and was ushered into existence by a midwife. It presented a large globular dilatation of the funis. The expansion commenced immediately at the insertion of the cord into the abdominal walls; it measured two and a half inches in its transverse diameter, and nearly the same in its long axis; the circumference of the narrowest part, or neck of the tumor, at the junction of the funicular tunics with the abdominal integuments, was nearly four inches; the skin around the umbilical aperture had been drawn down by the weight of the protrusion, forming an investment to its pedicle about half an inch long; the dilated funis evidently contained a portion of the hollow viscera of the abdomen, conveying impulses from that cavity, and pressure forcing air audibly from the incarcerated contents into the same.

The taxis was perseveringly tried, but without avail; and it was determined to expose and reduce the contents. The necessity for such a step was arrived at after mature deliberation, and on the following grounds: The expanded cord was in process of desiccation; in a few days its separation would expose the imprisoned contents; such process resulting either in adhesion of the intestine to the abdominal opening, with slough of the protrusion and the formation of a fæcal fistula, or fatal peritonitis would be the issue. The existing condition and probable results having been explained to the parents, they desired that any means might be used that would avert the formation of an unnatural outlet.

*Operation.*—An incision in the direction of the long axis was made through the funicular investment of the tumor; the dissection was carried carefully through all the tissues, until the intestine was exposed. It was impossible to detect any sac; the mass of contents was at every point adherent to the inside of the expanded funis. These adhesions were easily broken down, and the whole mass was liberated from its covering; but this simply exposed the contents, and further proceedings were necessary to enable us to reduce them. The whole had a truly formidable appearance; it had plainly been incarcerated for some time; it had none of the features of normal intestine; the surface of the folds was covered with organized lymph; the sulci, or involutions between the different folds, were filled up, level with the surface, with solid, semi-transparent lymph. The whole was gradually and carefully unfolded, the adhesions yielding readily; and, on examination, it proved to be the cœcum, a portion of the ascending colon, and a large portion of the ileum. It was now found that the opening through which this had passed, was too small to permit its return, so thickened and solidified had the intestinal coats become during their sojourn in the cord; the margin was notched on the right side to avoid the vessels, and the cutting instrument was guarded by the finger. A little gentle dilatation enlarged the opening sufficiently to admit of return of the gut, and a strong ligature, embracing the neck of integuments which surrounded the pedicle of the tumor, effectually closed the external opening. Over this a compress and bandage were applied, and the little patient was put to bed. The child did not appear to suffer much from such apparently harsh proceedings; the hemorrhage was not great, and came solely from vessels ramifying in the plastic material thrown out on the surface of, and in the interstices of the mass.

10th.—The child has been restless and cross through the night; the abdomen is slightly tympanitic; the bandage was removed, and tr. opii camph., gtts. ii., were ordered every hour.

11th.—Condition much the same as yesterday. Abdomen more distended, and resonant; the child does not take the breast, and vomits green mucosites; has had no evacuation by the rectum.

12th.—The patient remained in much the same state until night, when it died. Just before death, it had several watery stools.

13th.—Necroscopic examination eleven hours after death. The integuments are tinged of a yellow hue, abdomen tensely distended, and covered with large tortuous veins. The abdomen was opened by two elliptical incisions, inclosing the umbilicus; the inflated intestines bulged through the incisions, and, at one point, were found adherent to the ellipsis of skin removed in exposing the abdominal cavity. There was no effusion into the peritoneal sac, and the traces of inflammatory action were limited to those portions of the intestine that had been incarcerated in the funis; the descending, transverse, and a portion of the ascending colon, were found healthy, empty, and contracted; at about the middle of the ascending colon, was a well-marked contraction of the caliber of the gut, and immediately on the proximal side of this the intestine was expanded, and altered in structure. The expansion was not associated with thinning of the parietes of the tube; on the contrary, they were thickened in some situations to the extent of two or two and a half lines. This condition existed in the cœcum, and eighteen inches of the ileum, where it ceased less abruptly then at the seat of obstruction. The peritoneal surfaces of these portions of the tube were maroon colored, in some places purple, in others darker, approaching black; but they were not in a condition of sphacelus, were not softened, and did not give way at any part in the manipulation of examination. The mucous surface was less intense in color, but still highly congested, and its epithelium easily detached. The portions described were those that had formed the hernial tumor; the remaining portions of the intestine, the upper portion of the ileum, the jejunum, duodenum, and stomach, were greatly distended with aeriform and fluid contents.

### ERECTILE TUMOR.—VESICAL CALCULI.

This was an erectile tumor removed from the scalp. It made its appearance after the patient had received a blow on the head.

The third specimen was a *vesical calculus*, weighing six hundred and sixty grains, of the ammonio phosphatic variety. It was removed from a female fifty seven years old. The urethra was first dilated, the stone was crushed, and the fragments withdrawn with the forceps.

The fourth specimen was also a vesical calculus, of the uric acid variety, removed by the lateral operation, from a boy six years old. In two weeks he was well.

### RUPTURE OF THE AORTIC VALVE.

Dr. Sands exhibited a specimen of rupture of the aortic valve. It was removed yesterday from the body of a policeman.

*Post-mortem Examination.*—Sept. 14, 1859. 22 hours after death.

Rigor mortis marked. Body well developed. Skin tinged with yellow.

*Head.*—Not examined.

*Thorax.*—On raising the sternum and costal cartilages, both pleural sacs were found to contain a considerable quantity of recently effused serum. There was about a quart of this fluid in each pleura, pushing upwards the lungs, so that their lateral inferior margins corresponded with the fifth intercostal space. The lower lobe of each lung was compressed, dark colored, and did not crepitate on pressure; the remaining portions were healthy. No adhesions of either lung.

*Heart.*—On opening the pericardium, about an ounce of serum escaped. The heart was then removed, and presented externally the appearance of a healthy organ. Its cavities were next examined. Those of the right side were healthy and considerably distended with blood, partly fluid and partly in the form of yellowish coagula. On proceeding to open and expose the cavity of the left ventricle, very extensive disease was discovered of the semilunar valves of the aorta. Two of these had been ruptured; the rent extending through their thickness, and completely tearing up their attached convex borders, leaving, however, their concave free borders undisturbed. The remaining valve presented its normal aspect. Opposite one of the torn valves was the aortic sinus that gives origin to the left coronary artery; here the inner coat of the aorta was detached, and the subjacent tissues deeply stained with blood. Immediately below the line indicating the former attachment of this valve, was a small opening, through which a probe of ordinary size could be passed directly across from the cavity of the left ventricle into that of the corresponding auricle. On the auricular aspect of this opening was a smooth button of fibrine, about as large as a split pea, which had evidently been separated from the blood, as it had passed over the rough edges of the aperture during life. Fibrinous vegetations were also found in abundance upon all the roughened surfaces presented by the torn valves, and could easily be detached with the handle of a scalpel. The mitral valves were healthy. The arch of the aorta was studded with a few small patches of atheroma, but was otherwise free from disease. The heart weighed eleven and a half ounces.

*Abdomen.*—The subcutaneous adipose tissue was large in quantity, and of the same yellowish hue as the skin. The abdominal viscera were all healthy.

The PRESIDENT exhibited the following specimen, with the history of the case, on the part of a candidate for membership.

### DISEASE OF HEART AND CEREBRAL HEMORRHAGE.

The heart which I have the honor to present to the Pathological Society, was obtained August 12th, 1859. The case was seen by me the day previous, about an hour before death. The circumstances, so far as ascertained, were as follows: The patient was a stranger in the city, aged about 40, tall and spare. During the forenoon of the day of his death he seemed to be in comfortable health, was able to be out of doors, and, returning to his lodging-house between one and two o'clock, P. M., called for a luncheon of tea and toast. It was brought to him by a servant maid of the house, who left the room, and shortly returning, found him unconscious, his head resting on the table. He had eaten but a few mouthfuls. The only evidence of any approach toward a return of consciousness was that, in reply to a question whether he was better, he said, once, yes. He was seen by Dr. B. L. Budd a few moments after the attack. He was then completely unconscious, and the breathing was stertorous. The pulse at that time was extremely feeble, and the extremities were cold. Some brandy and water was administered without difficulty. When seen by me, about three quarters of an hour after the attack, stertor existed, and the respirations were irregular, but the pulse had considerable volume and force. The patient remained unconscious and perfectly motionless. The limbs were flaccid. The respirations became more and more irregular and unfrequent. The pulse soon became irregular, but retained considerable volume and force, even when the respirations occurred only three or four times per minute, the inspirations being short and spasmodic. The mode of dying was distinctly by apnœa. Death took place at about 3 P. M., somewhat less than two hours after the attack.

On applying the ear to the præcordia, when I first saw the patient, a bellows murmur was distinctly perceived accompanying the systolic sound. It appeared to be louder at the base than at the apex of the heart, but, under the circumstances, the examination was cursory, the clothes of the patient not having been removed. There was considerable systolic heaving of the præcordia. Visible pulsation of the carotids was marked. The aspect was notably pallid.

The previous history of the case is very imperfectly known. The patient was a produce merchant from Sandusky, Ohio. It was ascertained that he had an attack of rheumatism last winter, but not sufficiently severe to keep him within doors. For some time prior to his death he had not been well, but the nature of his ailments was not ascertained.

The examination *post mortem* was made, August 12, nineteen hours after death. The head was first examined. On removing the skull, a considerable quantity of extravasated blood, in clots, was situated within the arachnoid cavity upon the anterior lobes. It was most abundant on the right hemisphere. Pains were not taken to determine the quantity with exactness, but it must have amounted to between one and two ounces. A thin layer of coagulated blood, situated beneath the arachnoid membrane, extended over the greater part of the cerebrum, dipping down to the bottom of the convolutions. The arachnoid, pia mater, and stratum of coagulated blood were stripped off together with ease, leaving the surface of the brain remarkably white and firm. The brain substance was everywhere normal in appearance, excepting an unusual firmness and an anæmic aspect. No extravasation existed save at the periphery. No calcified arteries were discovered. The ventricles were empty.

The heart contained no clots nor fibrinous coagula. The cavities of the right side were distended with liquid blood. The weight of the organ, after the contents of the cavities were removed, was eleven ounces. The walls of the left ventricle were somewhat thickened, measuring at the thickest part five eighths of an inch. The cavity of the left ventricle did not appear to be dilated. The thickness of the walls of the right ventricle was not increased, and the cavity was not dilated. The aortic, pulmonic, and tricuspid valves presented nothing abnormal. The aorta was free from atheromatous deposit or other morbid changes. The muscular structure of the walls presented the appearance of healthy tissue. The deposit of fat upon the right ventricle was somewhat more abundant than usual. There were no traces of recent or old pericarditis. The form of the organ was normal.

Exclusive of a small amount of hypertrophy of the left ventricle, the morbid appearances which the heart presented were situated at the mitral valve and orifice. Two important tendinous cords attached to the inner portion of the anterior curtain of the mitral valve, were ruptured. The rupture had taken place at the points of attachment of the cords to the curtain. The ruptured cords remaining attached to the papillary muscle, must have floated freely within the cavity of the ventricle. Their movements during life had caused one of them to slip between a tendinous filament and a cord attached to the outer portion of the anterior curtain. This disposition was observed when the heart was first examined at the autopsy. The loose cords terminate in bulbous-like extremities, owing to the deposit of fibrin at the ruptured ends. The free border of the portion of the anterior curtain to which the ruptured cords had been attached, was studded with small excrescences irregular in size and form. Some of these have been removed in order to determine the firmness of their attachment. They were separated with moderate force, and appeared to be solidified fibrin. The remainder of the valve was free from morbid appearances. The auricular orifice was large, and evidently not freely protected by the valve. At the base of the valvular curtains, within the left auricle, were numerous small warty excrescences, some of which were detached with slight force. Over a space within the auricle of the the size of a dime, the endocardium was studded with a fine, granular deposit.

The lungs were entirely free from pleuritic adhesions, and presented a healthy appearance.

The liver was enlarged, but otherwise presented a normal appearance externally, and on section.

The stomach and intestines, inspected externally, presented nothing abnormal.

### ANEURISM OF THE ARCH OF THE AORTA.

Dr. Finnell presented a specimen of aneurism of the arch of the aorta. The case occured in a gentleman about fifty years old. He had suffered for ten years with a cough, pain in the chest, and difficulty of breathing, for which he placed himself under treatment, but with little benefit.

The patient died about four weeks ago; not *suddenly*, however, as usual in such cases, but gradually exhausted and worn out.

On post-mortem inspection, a bulging tumor was found beneath the sternum, which proved to be an aneurism. The three great vessels going off from the arch, diverged from the tumor; the left carotid was almost entirely obliterated, and the left subclavian somewhat diminished in size. The trachea was compressed by the tumor, and the œsophagus found to be in a sloughy condition.

Dr. Clark wished to know if the voice was affected.

Dr. Finnell stated that the voice was not altered, and that he did not suppose that the recurrent laryngeal nerve was pressed upon by the tumor. The patient had great difficulty in swallowing food.

### FORMATION OF ADIPOCIRE.

Dr. Dalton exhibited a remarkable specimen of *adipocire*, a body which had undergone complete transformation.

As far as could be ascertained, the body was buried in 1832. It was found in a cemetery, or rather a pit in the upper part of the city, which was dug out for the reception of cholera patients. The bodies were placed in separate coffins, but not in separate graves. The coffin containing this body was found about twenty feet beneath the surface; underneath it there were three tiers of coffins, and above it nine or ten. The uppermost tier of coffins was covered by three or four feet of solid earth. The soil directly under the coffin in which the body was found, was very watery; above this level there was but little water, although the ground was very moist. The bodies contained in this part of the pit were melted together in a semi-fluid mass, the usual result of decomposition under ordinary circumstances.

At the water-mark there were several bodies converted into adipocire. The specimen presented, however, was the most perfect. The hands and feet have been rattled off during transportation. When the body was first taken out, its color was almost precisely the same as now—a dullish white. If anything, it has become a little more brownish. It has now been exposed to the air for three months. Its consistency was decidedly less when first removed; it was then like cheese of medium consistency; a mixture of the ductile and the brittle. In handling it, great care had to be used. At that time, it exhaled a tolerably strong odor; partly cheesy, ammoniacal, and earthy. Since that time, the cheesy and the earthy odors have disappeared; the ammoniacal smell, however, is still perceptible. In other respects, it appears not to be altered in the least, and Dr. Dalton presumes it will remain in the same condition for years; for centuries, if properly taken care of.

The body is that of a large, fat woman, between forty-five and fifty years of age; evidently a woman past the prime of life. The anterior parietes have sunk very much, particularly those of the abdomen, which appear to be in contact with the spinal column. The anterior portion of the chest is also collapsed. The change of animal tissue to the adipocire is absolutely complete in all the tissues, except the hair, nails, and bones. The papillæ of the skin can be distinguished, but the other tissues cannot be made out.

The substance of which this mass is composed is known by the name of adipocire, or, as the French call it, "*gras des cadavres*" (fat of dead bodies). It is exceedingly light, so that one can easily raise the whole subject.

It is somewhat curious that all the bodies which are reported as having undergone this degeneration, have been interred under precisely the same circumstances. The first case was observed in a similar pit at a cemetery in Paris.

The chemical composition of the substance is such, that it is regarded as an ammoniacal soap; sometimes a soap composed of ammonia and lime, in other instances almost exclusively a lime soap. Orfila and Fourcroy, who paid particular attention to this subject, assert that at first it is almost exclusively ammoniacal; the ammonia being supplied by the decomposition of the nitrogenized muscular tissue. This unites with the fat coming from the adipose tissue, which has become rancid, and produces an ammoniacal soap. Some French chemists regard it as a transformation of the muscles into oleic acid ; so that adipocire may be produced by simple decomposition of the muscular tissue. The more generally received opinion is, that it is simple decomposition of the muscular tissues into ammonia, which unites with the fat of the adipose tissue. This opinion is favored by the fact, that in almost every instance of this kind the bodies are those of extremely fat persons. Such was the fact in a case, the only case of this kind which Dr. Dalton has previously seen, where the body was that of an enormously fat man. Another reason which makes it probable that the fat must come from the adipose tissue is, that, as Orfila ascertained, adipocire does not take place when the animal matter consists of muscular tissue only.

A body buried *by itself* will rarely be converted into adipocire, because the ammonia-compounds produced by the decomposition of the muscular substance, are dissolved in the fluids of the body, and these fluids absorbed by the soil, and do not unite with the fats so as to form adipocire. But if a body is surrounded by other bodies, the bodies above decomposing, produce ammoniacal fluids. These being washed down by the rain, filter through to the ninth or tenth coffin ; the water, of course, in its descent, becoming more and more loaded with ammonia, and this uniting with the fat of the lowermost bodies, produces adipocire. The bodies under the surface of the water do not undergo the transformation, probably because the substance is slowly soluble in water.

This material, of which the body is composed, is very inflammable. A piece put on charcoal, placed before the flame of the blowpipe, takes fire, and is consumed readily, leaving scarcely any appreciable residue.

---

Regular Meeting, Sept. 28, 1859. Dr. Finnell, Vice-Pres't, in the chair.

### INTRA-UTERINE POLYPUS.

Dr. J. Marion Sims presented a specimen of *intra-uterine polypus* which he thought was of some interest in a surgical point of view. The mass was attached to the fundus of the uterus near its anterior wall, and was of seven or eight years' growth. The subject was a colored woman, who came to the Woman's Hospital for treatment, when, by the introduction of the sponge tent, this pedunculated fibrous tumor was discovered. An operation for the removal of the tumor was delayed, inasmuch as the patient suffered from an attack of double tertian remittent fever, which lasted three weeks in spite of every treatment. In this connection, he remarked that he had often noticed that persons who had resided for a considerable length of time in the South (as was the case with this woman), and had not suffered from any miasmatic disease for a great while, and perhaps never, as soon as they came north, were seized with fever. He had been in the habit of cutting short such fevers by large doses of quinine, 20-grain doses by the stomach, and 40 by the bowels. This treatment was resorted to in this case, but the medicine could not be retained, and he was forced to give her mercurials; though the symptoms ceased at the end of three weeks, he was disposed to think, from its long resistance to treatment, that it was a self-limited disease, more like typhoid fever.

After the arrest of the symptoms, she was left in such a prostrated condition that he thought it yet imprudent to apply a ligature for fear of pyemia (if there is such a thing), or poison, by the disintegration of the tumor. At length, the patient having sufficient strength to bear up under an operation, he determined to use the ecraseur, a strong ligature being substituted for the chain. He stated that he had used this instrument (ecraseur) in the ordinary way for the removal of intra-uterine tumors four times, once in the presence of Dr. Metcalfe, and three times in the presence of Dr. Mott, and in each case experienced a great deal of difficulty in the proper adjustment of the chain. It was easy enough to apply the ligature, but not so with the chain, as it must work always at a certain angle. The ligature made use of was a strong fishing-line made of silk. The tumor was separated in the course of fifteen minutes by gradually turning the screw, the patient in the meantime under chloroform.

It was not possible to apply the ligature directly in contact with the inner surface of the uterus—perhaps half or three quarters of an inch distant. The recovery, notwithstanding, was complete, for he had ascertained two weeks after the application to the tumor that the little elevation left at the attachment entirely disappeared, and was as smooth as any other part of the surface of the organ.

In conclusion he remarked that he had found the application of the ligature to answer just as well as the chain; in fact he was disposed to think that it could be substituted for the chain in almost all cases.

### FEMORAL HERNIA.

Dr. Henry B. Sands exhibited a specimen of femoral hernia which occurred in the person of a lady of this city, fifty years of age. The history of the case was imperfect, but so far as could be ascertained was as follows: She was a lady of pretty good constitution, and generally enjoyed excellent health. She was, at the time of her death, on a visit to West Point, stopping at Cozzens' hotel. It was known that she was sufficiently well on Friday to take a carriage-drive, and it was also known that on the Sunday following she died. The prominent symptom in her case was vomiting, but the cause of death at the time was not known. Nothing more of the history is evident now except that she was attended during her whole illness by a surgeon of the army stationed at that place. The deceased was brought to this city, where Dr. Sands was called upon to make a post-mortem examination of the body.

*Post-mortem.*—The head was not examined. All the thoracic organs were found to be in a healthy condition. The cavity of the abdomen was next examined; the first appearance that was noticed was a considerable amount of injection of the parietal peritoneum and the peritoneum of the small intestines. It was also observed at the same time that the small intestine was very much increased in caliber, while the capacity of the colon was decreased to such an extent as to make it appear even smaller than the small intestine. A little further search revealed the cause of death: a femoral hernia which existed upon the left side.

The hernia was an exceedingly small one, a small portion of the ileum, eighteen inches from its termination, protruding in extent as here represented, and scarcely embracing the circumference of the gut. The intestine was pretty tightly knit, and the sac contained nothing else beside. The coats of the intestine at the protruded part were found to be considerably thickened, though they had not apparently lost any of their original strength. The cause of death could only be referred to *vomiting*, the inflammation of the peritoneum not being of sufficient severity. There could be seen a well-marked sulcus just above the point of constriction, which was caused by the accumulation of fæces which had taken place in that situation.

"Here," said he, "below the point of constriction, it will be noticed that the caliber of the intestine is very much reduced in size; the coats are very much thinned instead of being thickened, as is the case with the portion strangulated. The width of intestine above and below the hernia varies very greatly, below, measuring three quarters of an inch, and above, nearly an inch and half. This increase in caliber extended above as far as the duodenum, while the diminution in size extended downward through all the remaining portion of the canal.

As soon as this condition of parts was discovered, the very interesting question came up whether the existence of this state of things could have been ascertained before death? Before the gut was removed, an exceedingly careful examination was made in order to see if by any external sign the hernia could be recognized. The conclusion arrived at was, that it would be exceedingly difficult, and perhaps impossible, except by the existence of local tenderness. The obstacles at forming a conclusion were very great; not the least among these was the thickness of adipose tissue in the walls of abdomen, which, though not measured, was estimated at one and a half to two inches, while the same thickness of fat existed under the walls of the abdomen.

Percussion was resorted to, but no resonance elicited; the dullness being caused by the presence of the fatty tissue.

It could not be ascertained whether the surgeon's attention had been directed to the hernia as the cause of the symptoms; but it is pretty evident that a very careful surgeon might have overlooked it. Usually, when death occurs under such circumstances a great deal of blame is attached to the surgeon; but, knowing the gentleman to be a person of *great* capability, and being aware of the facts as shown by the post-mortem, it may serve to show that such a case would be very apt to be lost sight of by one most capable of finding it out.

### DISEASE OF THE KNEE JOINT.

Dr. T. C. Finnell presented a specimen of bones of the knee joint which had been the seat of a long-existing disease. It was taken from a patient who was operated upon in St. Vincent's Hospital about seven weeks before. The history of the case as given by the patient was, that he had injured the knee five years ago by a fall, which was followed by acute inflammation in the part, which lasted about six weeks, at the end of which time he was able to get about; yet he did not entirely recover. During the three years which followed, he was the subject of repeated attacks of inflammation in the part, which were however not very severe, but caused him for the time being to remain quiet a few days. All this time the symptoms of disease increased, causing him more and more discomfort until two years ago, when motion of the joint was so painful that he was forced to nurse it constantly and remain in doors. About this time several sinuses formed, which were found to communicate with the cavity of the joint. As a consequence of all this, the disease continuing, his general system began to suffer, and at the time he entered the hospital, he was in a very feeble condition, suffering from night-sweats, cough, &c.

A consultation was called, and the question of exsection of the joint came up for consideration; but it was not deemed practicable, inasmuch as his system was not only very much below par, but the condition of the joint was such as to leave but little hope that the operation would be attended by any wished-for result. There was bare bone to be felt not only at the ends of the bones, but at least an inch above and below the joint; this condition of things also existed in the popliteal space; beside all this, the neighboring parts had become so much disintegrated that there was hardly any sound tissue to be discovered. We were thus narrowed down to the expediency of amputation; which being decided upon, was performed. From that time the general health of the patient improved, and he made a rapid recovery.

On examining the bones before maceration, the cartilages of incrustation were found to have been almost entirely destroyed, some few points only remaining healthy. The limb was anchylosed in the straight position by the exudation in the tissues around the joint. The crucial ligaments were entirely destroyed, and those portions of the bones in contact with each other were carious. The articulating surface of the patella was entirely deprived of cartilage. The sinuses alluded to communicated with large abscesses which surrounded the joint, and one of these communicated with the cavity of the joint proper. At one point the periosteum was detached fully three inches from the lower end of the femur, by purulent fluid.

There was not much pus in the cavity of the joint, the bones being in close apposition to each other.

